# Electrolyte Imbalance in Children With Severe Acute Malnutrition at a Tertiary Care Hospital in Pakistan: A Cross-Sectional Study

**DOI:** 10.7759/cureus.10541

**Published:** 2020-09-19

**Authors:** Mohammad Raza, Sohail Kumar, Muzamil Ejaz, Dua Azim, Saad Azizullah, Azhar Hussain

**Affiliations:** 1 Pediatrics, The Indus Hospital, Karachi, PAK; 2 Pediatrics, Dow Medical College, Dr. Ruth K. M. Pfau Civil Hospital, Karachi, PAK; 3 Internal Medicine, Dow Medical College, Dr. Ruth K. M. Pfau Civil Hospital, Karachi, PAK; 4 Healthcare Administration, Franklin University, Columbus, USA; 5 Medicine, Xavier University School of Medicine, Oranjestad, ABW

**Keywords:** severe acute malnutrition, electrolyte imbalance, children, hypokalemia, hypocalcemia, hyponatremia, hypomagnesemia

## Abstract

Background

Malnutrition is a significant public health concern and a leading contributor to the global burden of children’s diseases, affecting 50 to 150 million children under the age of five years worldwide. Globally, undernutrition accounts for approximately 33% of the deaths among under-fives. South Asia alone contributes to 50% and 38.8% of the world’s population of wasted and stunted children, respectively. In Pakistan, malnutrition is the leading cause of childhood mortality, accounting for nearly 35% of all deaths under five years of age. Severe acute malnutrition (SAM), the most severe form of malnutrition, is often associated with electrolyte imbalances. This study aimed to determine the frequency of electrolyte imbalance in children with SAM admitted at a tertiary care hospital.

Methods

This cross-sectional study includes 184 patients with SAM aged between 6 and 60 months, who were admitted at the inpatient Department of Pediatrics, Dr. Ruth K. M. Pfau, Civil Hospital, Karachi, Pakistan, from January 17, 2017 to July 16, 2017. Weight and length/height were measured, and weight-for-height was calculated. Children were labeled to have SAM when weight-for-height was below -3 standard deviation (SD). Blood samples for serum electrolytes were drawn and sent to the lab. Descriptive statistics were calculated, and stratification was performed using the chi-square test. A p-value of ≤0.05 was considered statistically significant.

Results

The mean age of participants was 22.63 ± 12.71 months. Of the 184 patients with SAM, 172 (93.5%) patients had electrolyte imbalance. Hypokalemia was present in 79.9%, whereas hypocalcemia, hyponatremia, and hypomagnesemia were present in 71.7%, 48.9%, and 13.6%, respectively. Post-stratification results showed a significant association of electrolyte imbalance with gender (p = 0.005) and educational status of parents (p = 0.001).

Conclusions

Electrolyte disturbances are common in SAM. Serum electrolytes of every malnourished child admitted should be assessed and corrected to avoid fatal outcomes. We suggest that more research with better study designs should be conducted to develop policies and strategies for successfully combating malnutrition in Pakistan. In the meantime, we recommend adopting national guidelines for the management of acute malnutrition to reduce morbidity and mortality.

## Introduction

Malnutrition entails an imbalance between the supply of nutrients and energy and their demand by the body for adequate growth and development. Malnutrition is expressed in many forms, including undernutrition, stunting in under-fives, wasting in under-fives, micronutrient deficiencies, being overweight in adults, and obesity in adults [1]. Malnutrition is a significant public health concern and a leading contributor to the global burden of children’s diseases. Globally, around 50 to 150 million children under the age of five years are malnourished. According to the United Nations Children's Fund (UNICEF), poor nutrition accounts for one-third of the deaths among under-fives [2]. South Asia, regarded as an “Asian Enigma”, is the most severely afflicted area due to poor hygienic conditions, high rates of low birth weight, unsatisfactory breastfeeding practices, and poor socioeconomic status of women. According to the World Health Organization (WHO), South Asian countries, namely Bangladesh, India, and Pakistan, constitute approximately 50% and 38.8% of the world’s population of wasted and stunted children, respectively [3]. In Pakistan, malnutrition contributes to nearly 35% of all death under five years of age. According to the National Nutrition Survey 2011, 31.5% of all children were underweight, almost 43% had stunted growth and 15.1% were suffering from wasting [4].

Severe acute malnutrition (SAM) is the most severe form of undernutrition. Malnourished children often have associated electrolyte imbalances. Electrolyte imbalance, when accompanied by diarrheal diseases, has a multiplicative effect on the risk of childhood morbidity and mortality [5,6]. In severely malnourished edematous conditions, most children have excess total body sodium (Na) despite low serum sodium levels; low levels of serum sodium, thus, masks the sodium overload [5,7]. Total body potassium (K), calcium (Ca), and magnesium (Mg) are also depleted, even at normal serum levels [7,8].

Hypokalemia often presents with muscle weakness, hypotonia, apathy, paralytic ileus, and cardiac arrhythmias [5]. Clinical characteristics of hypocalcemia are often subtle. However, hypocalcemia can lead to fatal seizures in children when accompanied by hypomagnesemia [8]. Magnesium is essential for bioenergetic reactions, membrane stabilization, and nerve conduction; its deficiency can cause convulsions and cardiac arrhythmias [9].

According to previous studies, hyponatremia and hypokalemia are the common electrolyte disturbances in malnourished children. The frequency of the said electrolyte disturbances increases in the setting of diarrhea [5,10,11]. The rationale behind this study is to estimate the burden of electrolyte imbalance in children with SAM taking Na, K, Ca, and Mg as components of electrolytes to establish strategies for prompt treatment and to prevent lethal complications in such children. Although there are multiple studies, local as well as international, on electrolyte imbalance in SAM, only a few electrolyte components were studied; some evaluated Na and K levels only, whereas others assessed Na, K, and Ca levels.

## Materials and methods

Study design and patient population

We conducted a hospital-based cross-sectional study at the inpatient Department of Pediatrics, Dr. Ruth K. M. Pfau, Civil Hospital, Karachi, Pakistan, from January 17, 2017 to July 16, 2017. Our study included 184 children aged between 6 and 60 months suffering SAM for three months or less. The sample size was calculated using the WHO sample size calculator 1.1. A sample size of 184 children with the allowable margin of error of 6.5% and the confidence level of 95% was calculated based on the prevalence rates of hypomagnesemia in malnourished children from a previous Indian study [9]. This study was regulated by the Research, Training and Monitoring Cell of College of Physician and Surgeons, Pakistan. Written informed consent was obtained from the mother/caregiver of every child before their enrollment in the study.

Children with secondary causes for wasting, such as congenital heart disease, chronic kidney disease, chronic liver disease, cerebral palsy, celiac disease, disseminated tuberculosis, malignancy, and hemolytic anemia, were excluded. The secondary causes were ruled out based on the diagnosis made through history, physical examination, and relevant lab investigations.

Data collection

Each child was assessed by taking a detailed history from the mother/caregiver and by performing a physical examination including anthropometry. Weight and length/height were measured, and weight-for-height was calculated. Length/height was measured using a stadiometer, and weight was measured using a standard weight machine. Children were labeled to have SAM when weight-for-height was below -3 standard deviation (SD) using the WHO child growth standards [12]. Basic demographic information such as name, age, gender, and address was recorded. A blood sample for serum electrolytes was drawn by a senior staff under all aseptic measures and sent to Civil Hospital Karachi Central Lab. Non-probability consecutive sampling was employed. All the information was recorded on a specified pro forma.

The frequency of electrolyte imbalance was determined based on the following four parameters; if a patient had a deficiency of one or more of the electrolytes listed below then that patient was labeled as having electrolyte imbalance:

1. Hyponatremia: when serum sodium is <136 mEq/L.

2. Hypokalemia: when serum potassium is <3.5 mEq/L.

3. Hypocalcemia: when serum calcium is <8.4 mEq/L.

4. Hypomagnesemia: when serum magnesium is <1.6 mEq/L.

These cut-off values were taken according to that set by Civil Hospital Karachi Central Lab.

Statistical analysis 

Collected data were analyzed using Statistical Package for the Social Sciences (SPSS) version 22.0 for Windows (IBM Corp., Armonk, NY, USA). Mean ± SD was calculated for quantitative variables such as age, the serum concentration of Na, K, Ca, and Mg, and the duration of SAM. Percentages were calculated for qualitative variables such as the general frequency of electrolyte imbalance in children with SAM, as well as separate frequencies and percentages for hyponatremia, hypokalemia, hypocalcemia, and hypomagnesemia, and educational status of parents. Effect modifiers such as age, duration of SAM, gender, and educational status of parents were controlled by stratification. The results were presented in the form of tables. The post-stratification chi-square test was applied to observe the effect of modifiers on the outcome. A p-value of ≤0.05 was considered statistically significant.

## Results

Out of 184 children, 62% (n = 114) were males and 38% (n = 70) were females. The mean age of children was 22.63 ± 12.71 months, and the mean duration of SAM was 44.13 ± 24.93 days. The mean serum levels of Na, K, Ca, and Mg are shown in Table 1.

**Table 1:** Mean ± SD values of serum electrolyte levels (N = 184) SD, standard deviation *Range of serum electrolyte levels within the sample population

Variables	Mean ± SD	Range*
Serum sodium (mEq/L)	135.65 ± 4.27	129.0-143.0
Serum potassium (mEq/L)	2.89 ± 0.799	1.7-4.8
Serum calcium (mEq/L)	7.70 ± 1.03	6.0-9.9
Serum magnesium (mEq/L)	1.93 ± 0.43	0.9-3.0

Of all the parents interviewed, 35.9% were illiterate, whereas 43.5%, 16.8%, and 3.8% had primary, secondary, and intermediate or above level education, respectively. Our analysis showed that 93.5% of children had electrolyte imbalances. Most of them had hypokalemia accounting for about 79.9%, whereas hypocalcemia, hyponatremia, and hypomagnesemia were present in 71.7%, 48.9%, and 13.6% of the children, respectively, as shown in Table 2.

**Table 2:** Frequency distribution of electrolyte imbalance (N = 184)

Variables	Present	Absent
	n (%)	n (%)
Hyponatremia	90 (48.9)	94 (51.1)
Hypokalemia	147 (79.9)	37 (20.1)
Hypocalcemia	132 (71.7)	52 (28.3)
Hypomagnesemia	25 (13.6)	159 (86.4)

Stratification concerning gender, age, duration of SAM, and the educational status of parents was performed to observe the effect of these modifiers on the outcome (electrolyte imbalance). A p-value of ≤0.05 was considered significant. The results showed a significant association of electrolyte imbalance with gender (p = 0.005) and the educational status of parents (p = 0.001). No significant association was found with age (p = 0.154) and duration of SAM (p = 1.000). The detailed results are presented in Table 3.

**Table 3:** Stratification with respect to gender, age, duration of SAM, educational status of parents, and electrolyte imbalance SAM, severe acute malnutrition *p-value of ≤0.05 was considered statistically significant

	Electrolyte Imbalance			p-Value
	Yes (N = 172)	No (N = 12)	Total (N = 184)	
	n (%)	n (%)	n (%)	
Gender				0.005*
Male	102 (59.3)	12 (100)	114 (62)	
Female	70 (40.7)	0 (0)	70 (38)	
Age (months)				0.154
≤24	120 (69.8)	6 (50)	126 (68.5)	
>24	52 (30.2)	6 (50)	58 (31.5)	
Duration of SAM (days)				1.000
≤30	86 (50)	6 (50)	92 (50)	
>30	86 (50)	6 (50)	92 (50)	
Educational status of parents				0.001*
Illiterate	66 (38.4)	0 (0)	66 (35.9)	
Primary	68 (39.5)	12 (100)	80 (43.5)	
Secondary	31 (18)	0 (0)	31 (16.8)	
Intermediate or above	7 (4.1)	0 (0)	7 (3.8)	

## Discussion

Electrolyte disturbances are common in malnutrition and continue to be a major health challenge in developing nations. In malnutrition, serum electrolyte levels represent the circulating concentration of electrolytes rather than the whole body content. In our study, 93.5% of children admitted with SAM had electrolyte imbalance.

Hypokalemia, present in approximately 79.9% of the patients, was found to be the most common electrolyte abnormality in our study. Similar results have been observed in previous studies; Zulqarnain et al. [6] reported hypokalemia in 61.1% cases of malnourished children, whereas Memon et al. [5] reported hypokalemia in 48% of the cases. Memon et al. [5] also noted that hypokalemia was more common in those who had malnutrition with diarrhea (62.5%) compared to those who had malnutrition without diarrhea (22.22%). Another research conducted by Gangaraj et al. [11] revealed the prevalence of hypokalemia in 61.22% (30/49) of malnourished children with diarrhea and vomiting and 33% (9/24) of malnourished children without diarrhea and vomiting.

Clinically significant hyponatremia was present in 48.9% of the children with SAM in our study. These results are comparable to the study by Gangaraj et al. [11] in which hyponatremia prevailed in 40.8% of cases with diarrhea and 14.8% of cases without diarrhea. In addition, Zulqarnain et al. [6] reported a prevalence of 31.1% of hyponatremia, whereas Memon et al. [5] observed hyponatremia in 26.56% and 13.88% of patients with and without diarrhea, respectively. Contrary to our findings, Fatima et al. [13], Bilal et al. [14], and Kamberi et al. [15] reported a lower frequency of hypokalemia and hyponatremia. In a cross-sectional study conducted at the National Institute of Child Health (NICH), Sameen and Moorani [10] also reported a lower prevalence of hyponatremia (22.6%) and hypokalemia (13.7%) as compared to our results; however, hyponatremia and hypokalemia were still among the most common metabolic abnormalities found in children (age < 5 years) with SAM [10].

An interesting finding of our study was the increased prevalence of hypocalcemia (71.7%) and the decreased prevalence of hypomagnesemia (13.6%). In contrast to our results, Zulqarnain et al. [6] reported hypocalcemia in only 13.3% of malnourished children. In a case-control study conducted in Bangladesh, Chisti et al. [8] reported a 26% prevalence of hypocalcemia among severely malnourished under-five children, whereas hypomagnesemia was noted in only 3.3% cases. In an Indian study, hypomagnesemia was present in 27% (17/62) of the malnourished children [9].

In this study, we noted that the mean level of serum Na was 135.65 ± 4.27 mEq/L and that of serum K was 2.89 ± 0.799 mEq/L, which were similar to the results by Shaheen et al. [16] who revealed mean serum Na and K values to be 133.90 ± 3.31 mEq/L and 3.25 ± 1.47 mEq/L, respectively. The mean age of children in our study was 22.63 ± 12.71 months, which was very similar to that reported by Fatima et al. [13] (23.56 ± 3.80 months) and Bilal et al. [14] (1.9 ± 1.4 years); however, it differed from the result of Zulqarnain et al. [6] (3.28 ± 1.2 years).

Out of 184 children in our study, 114 (62%) were males and 70 (38%) were females. Several other studies also showed a similar male-dominant pattern. There were 62% (62/100) males in a study by Fatima et al. [13], 57% (57/100) males in a study by Memon et al. [5], 61.3% (49/80) males in a study by Bilal et al. [14], and 64.4% (58/90) in a study by Zulqarnain et al. [6]. The possible explanation for apparent male predominance is unknown. Of all the parents interviewed in this study, 35.9% were illiterate, whereas 43.5%, 16.8%, and 3.8% had primary, secondary, and intermediate or above education, respectively. Studies from Pakistan, Vietnam, Ethiopia, and India also revealed the educational status of the parents to be significantly less among children with SAM [16-20]. Illiterate parents are unaware of the composition of balance required for their child’s normal growth. Thus, an inappropriate diet lacking essential nutrients results in serum electrolyte imbalance.

To the best of our knowledge, this is Pakistan’s first study to assess the prevalence of electrolyte imbalances in children with SAM taking Na, K, Ca, and Mg into consideration at the same time. Although similar studies from Pakistan concerning electrolyte disturbances in malnourished children exist, they either did not address SAM specifically or studied only a few components of serum electrolytes.

There are certain limitations to this study. This research was a single hospital-based study. The principal limitation of this study is a non-randomized study design. Moreover, it had a small sample size; therefore, the results might not be generalized to larger populations.

## Conclusions

Electrolytes disturbances are common in children with SAM. We found hypokalemia followed by hypocalcemia as the common electrolyte abnormalities in children with SAM. The association of electrolyte imbalance with male gender and lower educational status of parents is also noteworthy. In contrast to already published literature on SAM, we observed a higher prevalence of hypocalcemia and a relatively lower prevalence of hypomagnesemia. Hence, we advise that serum electrolytes of every malnourished child admitted should be evaluated and corrected at the earliest to avoid any life-threatening outcomes. There is also a need to start a mass campaign to educate parents regarding the challenge of malnutrition. The development of policies and strategies for successfully combating malnutrition in Pakistan is only possible through unified statistics from all regions of Pakistan. We suggest that more research with better study designs in terms of the nature of the study, sample size, targeted regions, and varied age groups should be conducted to explore all factors contributing to malnutrition in Pakistan. In the meantime, we recommend adopting national guidelines for the management of acute malnutrition to reduce morbidity and mortality.
